# MicroRNA-144 inhibits the metastasis of gastric cancer by targeting MET expression

**DOI:** 10.1186/s13046-015-0154-5

**Published:** 2015-04-17

**Authors:** Jun Liu, Hui Xue, Jianjun Zhang, Tao Suo, Yijin Xiang, Wen Zhang, Jun Ma, Dingfang Cai, Xixi Gu

**Affiliations:** Department of Integrative Medicine, Zhongshan Hospital, Fudan University, Shanghai, PR China; Shanghai University of Traditional Chinese Medicine, School of Basic Medicine, institution of Neijing, Shanghai, 201203 China; Department of General Surgery, Zhongshan Hospital, Fudan University, Shanghai, PR China; Department of Oral and Maxillofacial-Head & Neck Oncology, Ninth People’s Hospital, Shanghai Jiao Tong University School of Medicine, Shanghai, 200011 PR China

**Keywords:** microRNA, miR-144, MET, Gastric cancer, Metastases

## Abstract

Gastric cancer (GC) remains one of the most common types of malignant cancer, and the molecular mechanism underlying its metastasis is still largely unclear. MicroRNAs have emerged as important regulators of metastasis because of their ability to act on multiple signaling pathways. In our study, we found that miR-144 is significantly downregulated in both highly metastatic GC cell lines and tissues. Results from both gain-of-function and loss-of-function experiments demonstrate that increased miR-144 expression significantly reduced GC cell migration, whereas decreased miR-144 expression dramatically enhanced GC cell migration. The met proto-oncogene (MET), which is often amplified in human cancers and functions as an important regulator of cell growth and tumor invasion, was identified as a direct target of miR-144. Moreover, silencing of MET using small interfering RNA (siRNA) recapitulated the anti-metastatic function of miR-144, whereas restoring MET expression attenuated the function of miR-144 in GC cells. Furthermore, we found that miR-144, by targeting MET, suppresses phosphorylation of Akt. Finally, we observed an inverse correlation between the expression of miR-144 and MET mRNA in GC metastatic tissues. In summary, miR-144 suppresses GC progression by directly downregulating MET expression, which subsequently prevents activation of the pro-oncogenic Akt pathway. Reintroduction of miR-144 expression in GC cells presents an attractive therapeutic approach to block the metastasis of gastric cancer.

## Introduction

Globally, gastric cancer (GC) is one of the most prevalent types of malignant disease. In 2008, approximately 989,600 new cases of GC were diagnosed. Furthermore, GC was implicated as a cause of 738,000 deaths, making GC the fourth most common malignancy and the leading cause of cancer death worldwide [1]. As the tumor progresses, it develops the ability to invade surrounding tissues and metastasize. The hepatocyte growth factor receptor, MET, is known to promote the motility and invasive capability of tumor cells [2]. MET is a member of the receptor tyrosine kinase family and has been shown to be upregulated in many tumors [3-5]. It is suggested that MET expression level is increased by either gene amplification or hypoxia via HIF1α. In patients with metastatic GC, MET amplification and strong protein expression are not infrequent. These events appear to be significantly associated with unfavorable clinical outcome [6]. Approximately 10% of white patients harbor a gain of five or more copies of MET. Additionally, this gain in MET copy number is significantly associated with unfavorable prognosis [7]. Furthermore, the miR-34a/c microRNAs have been shown to negatively modulate MET expression in cell lines derived from prostate cancer, hepatocellular carcinoma and GC [8-10].

MicroRNAs (miRNAs) are non-coding RNA molecules, approximately 21–23 nucleotides in length, which regulate gene expression at the transcriptional or post-transcriptional level [11-13]. miRNA expression profiling analyses have revealed a global downregulation of mature miRNA levels in human tumors relative to normal tissues [14]. Furthermore, miRNAs may function in either a tumor suppressor or oncogenic role, depending on the function of their target. For example, miR-133b was significantly down-regulated in GC tissues and exerted its tumor suppressor role in GC cells [15]. Expression of miR-337-3p was significantly downregulated in lymph node metastatic tissues of GC patients, and induction of miR-337-3p expression did reduce gastric cancer cell invasion capacity [16]. miR-25 promotes GC progression by directly downregulating TOB1 expression; therefore, increased expression of miR-25 presents a potential noninvasive biomarker for the prognosis of GC patients [17]. Additionally, miR-7 is significantly downregulated in both highly metastatic GC cell lines and metastatic tissues. Overexpression of miR-7 markedly inhibits GC metastasis by targeting the expression of the insulin-like growth factor-1 receptor (IGF1R) oncogene [18]. In GC cell lines, reintroduction of miR-144 expression results in repression of ZFX, which moderately increases cancer cell susceptibility to 5-fluorouracil chemotherapy. In 93 cases of primary GC, diminished miR-144 expression was associated with poor prognosis [19]. These examples have highlighted the key role of miRNAs in GC malignance and cancer progression.

In this study, we characterized the targets and defined the mechanism of action of miR-144 in GC. By inducing ectopic expression of miR-144, we discovered MET is a novel target of miR-144 regulation. This finding was confirmed on both mRNA and protein levels, and reporter gene luciferase assays verified direct binding of miR-144 to the regulatory binding site in the 3′UTR of MET. Furthermore, we found that MET expression inversely correlates to miR-144 levels in a small but well-documented GC cohort. We hypothesize that miR-144 inhibits GC metastasis, and that some of this inhibition is mediated by targeting MET expression.

## Materials and methods

### Human tissue specimens and cell lines

GC samples were collected from patients who underwent surgery at Fudan University Shanghai Cancer Center between 2012 and 2013. The protocol was approved by the Clinical Research Ethics Committee of Fudan University, and the research was carried out according to the provisions of the Helsinki Declaration of 1975. All samples were obtained with the informed consent of the patients. The human GC cell line AGS (ATCC® CRL-1739™), SNU-1 (ATCC® CRL-5971™), SNU-5 (ATCC® CRL-5973™), SNU-16 (ATCC® CRL- 5974™), NCI-N87 (ATCC® CRL-5822™), and KATO III (ATCC® HTB-103™) were maintained in DMEM containing 10% fetal bovine serum. All cell lines were maintained in media containing penicillin (100 IU/ml) and streptomycin (100 mg/ml) at 37°C with 5% CO2. The miRNA mimics and inhibitors were purchased from Ambion (Austin, TX, USA).

### RNA extraction and real-time PCR

Total RNA was extracted from cells using TRIzol (Invitrogen, Carlsbad, CA). For miRNA analysis, poly(A) tails were added to total RNA using poly(A) polymerase (Ambion, Carlsbad, CA) prior to reverse transcription. The MiRcute miRNA qPCR detection kit (TIANGEN, Beijing, China) was used to quantitate the expression levels of miR-144 according to the provided protocol. The following PCR conditions were used: 95°C for 30 s, followed by 40 cycles of 95°C for 5 s and 60°C for 31 s. The amount of target (MET/miR-144), normalised to the endogenous housekeeping gene GAPDH/U6snRNA and relative to a reference sample, is given by the following equation: amount of target =2-△△CT.

### Microarray hybridization

Briefly, RNA samples were used to synthesize double-stranded complementary DNA (cDNA), and double-stranded cDNA was labeled and hybridized to the Microarray (Arraystar, Rockville, MD). After hybridization and washing, processed slides were scanned with the Axon GenePix 4000B microarray scanner (Molecular Devices, Sunnyvale, CA). P value was calculated using the paired t-test. The threshold set for up- and down-regulated genes was a fold change > 2.0 and a p value < 0.05. Hierarchical clustering was performed based on differentially expressed genes and miRNAs using Cluster Treeview software from Stanford University (Palo Alto, CA).

### MiRNA-gene network

We constructed the network adjacency between two genes, i and j, defined as a power of the Pearson correlation between the corresponding gene-expression profiles, xi and xj. The adjacency matrix, M (i,j), was visualized as a graph, and the topological properties of this graph were examined. To make a visual representation, only the strongest correlations (>0.98) were drawn in these renderings. In miRNA-gene networks, each gene corresponds to a node. Two genes are connected by an edge, indicating a strong correlation. Within the network analysis, a degree is the simplest, most important measure of the centrality of a gene within a network and determines the relative importance. A degree is defined as the number of directly linked neighbors.

### Prediction of miR-144 binding site

Putative miR-144 binding sites in MET mRNA 3′ untranslated region were predicted by the Target Scan program (http://www.targetscan.org). Position 1430–1436 of MET 3′ UTR has a conserved binding site for miR-144 targeting.

### Plasmid transfection

The ORF sequences of MET were amplified from genomic DNA isolated from the SNU-5 cell line and were then subcloned into the plenti vector. The plasmid was transfected into SNU-5 cells using Lipofectamine 2000 (Invitrogen). After 24 h, the cells were used for a rescue experiment.

### Oligonucleotide transfection

MiR-144 mimics, miR-144 inhibitor (anti–miR-144), and MET siRNA (siRNA-MET) were synthesized by Genepharma, Shanghai, China. Oligonucleotide transfection was performed with Lipofectamine 2000 reagents (Invitrogen, Carlsbad, CA, USA). The final concentration of miR-144 mimics, anti–miR-144 or siRNA-MET in the transfection system was 100 nM. Transfection efficiency for the single and co-transfected studies was determined by fluorescence microscope.

### Immunoblotting

Equivalent amounts of cell lysates were resolved by 7% SDS/PAGE and were transferred onto polyvinylidene fluoride membranes. The membrane was incubated with a rabbit polyclonal anti-MET antibody (1:500, abcam, ab47431), a goat polyclonal anti-ADAM12 antibody (0.3 μg/ml, abcam, ab28747), and a rabbit polyclonal anti-Versican antibody (1 μg/ml, abcam, ab19345). IRdye-labeled secondary antibodies were used for quantitation of the immunoblotting signal, and the signals were analyzed using an Odyssey scanner (LI-COR Biosciences, Lincoln, NE, USA).

### RNA-ChIP assay

RNA-protein interactions are fixed with formaldehyde, and chromatin shearing is combined with DNase treatment to yield RNA/protein complexes that can be immunoprecipitated with antibodies to MET proteins. Cross-links are subsequently reversed; RNA is recovered and again treated with DNase to ensure the absence of DNA. RNA precipitated from the immune complex then be analyzed by real-time PCR. In this RNA-ChIP assay, the following formula be used: % of input (recovery) = AE(Ct input-Ct sample) * Fd * 100%. Here, AE is the amplification efficiency (10(−1/slope)) and Fd is a dilution factor of the input RNA to balance the difference in the amounts of RNA-ChIP sample and input RNA used for real-time PCR.

### Luciferase assay

The full MET 3′UTR was amplified by PCR using SNU-5 cDNA as template and cloned into pGL3 control vector. We used Quick Change mutagenesis to mutate the miR-144 putative binding site (Stratagene, Santa Clara, CA, USA). SNU-5 cells and AGS cells were transfected with miR-144 mimics/inhibitors and pGL3 luciferase reporter constructs harboring the MET 3′UTR. After 24 h, the activities of firefly luciferase and renilla luciferase in the cell lysates were measured with the Dual-Luciferase Assay System (Promega, Madison, WI, USA).

### Migration assays

For the transwell migration assays, 1 × 105 cells were plated in the top chamber containing a non-coated membrane. The cells were plated in the serum-free medium, and medium supplemented with 10% (v/v) serum was used as a chemoattractant in the lower chamber. The cells were incubated at 37°C in a tissue culture incubator with 5% (v/v) CO2. After 16 h, the non-migrated cells were removed from the upper sides of the transwell membrane filter inserts. The migrated cells on the lower sides of the inserts were stained with coomassie brilliant blue, and the cells were counted.

### Cell proliferation assay

The transfected cells were seeded in 96-well plates at a density of 1 × 104 cells/well. A cell proliferation assay was performed using the Cell Counting Kit-8 (Dojindo, Kumamoto, Japan) according to the manufacturer’s instructions. Before the addition of CCK-8, the cells were washed with warm culture media by spinning the plate at 500 rpm for 3 m and then discarding the supernatant.

### Primers

The following primers were used for real-time PCR: miR-144: 5-TACAG TATAG ATGAT GTACT-3; U6snRNA: 5-CGCAA GGAUG ACACG CAAAU UCGUG AAGCG UUCCA UAUUU UU-3; SKIL forward primer: 5-GTTAA GCGAA CCTGT ACTTC TGT-3, reverse primer: 5- GTAGG CGACA TGCTT TCTTG G-3; MET forward primer: 5-GTCGG AGTAG AGCGT CGAGA-3, reverse primer: 5-CAGCG CGATC AGGTA GAGC-3; TOP2A forward primer: 5-ACCAT TGCAG CCTGT AAATG A-3, reverse primer: 5-GGGCG GAGCA AAATA TGTTC C-3; ADAM12 forward primer: 5-TCAAC CTGGA TACCC GATTC C-3, reverse primer: 5-GCTCT GTCTG CCGAT GGAG-3; VCAN forward primer: 5-GTAAC CCATG CGCTA CATAA AGT-3, reverse primer: 5-GGCAA AGTAG GCATC GTTGA AA-3. The following primers were used for full MET 3UTR application: MET 3′UTR forward primer: 5-TCACT GCCTG ACCTT TA-3, MET 3′UTR reverse primer: 5-ATCAC TTACT CCCAC AAT-3. The siRNA nucleotide for MET was used as follows: siRNA-MET forward: 5-GUGCC ACUAA CUACA UUUAU U-3, siRNA-MET reverse: 5-UAAAU GUAGU UAGUG GCACU U-3.

### Statistical analysis

The results are presented as means ± SEM, and the data were analyzed with Student’s t test. A value of p < 0.05 was considered statistically significant.

## Results

### MicroRNA expression profile in gastric cancer

**Figure 1:**
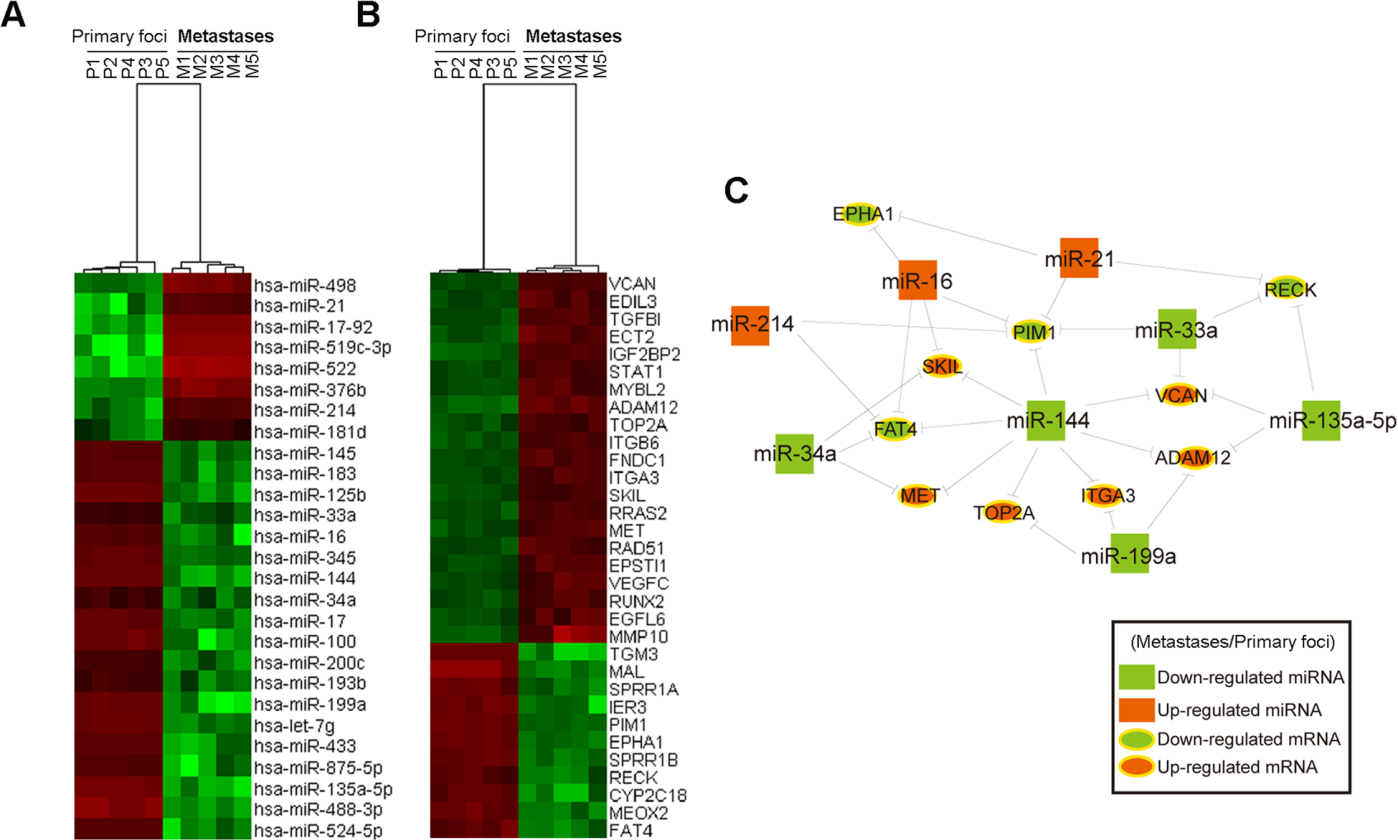
Core miRNA-gene network, including 8 key miRNAs and their targets. Hierarchical clustering analysis of 27 miRNAs **(A)** and 32 genes **(B)** that were differentially expressed between metastatic tissues in the peritoneal and paired primary samples of GC (greater than 2.0-fold; p < 0.05). Expression values are represented in shades of red and green, indicating expression above and below the median expression value across all samples. **(C)** The miRNA-gene network shows the relationships between 8 key miRNAs and tumor-associated genes they are predicted to regulate. The colors indicate the annotated expression levels of the miRNAs and genes.

Comparison of peritoneal metastatic tissues with paired primary foci samples of GC using hierarchical clustering analysis revealed systematic variation in the expression of miRNAs and genes (Figure 1A and B). Our data suggests that a set of miRNAs and genes is frequently aberrantly expressed in the peritoneal metastatic tissues of GC. In addition, we also found that some previously well proved molecules, such as miR-7 [18], miR-25 [17], TOB1 and IGF1R, were not be identified in our microarrays. We thought these difference may be induced by diversity of clinical samples came from different areas.

The miRNA-gene-network was assembled with the goal of identifying the key miRNAs that regulate tumor-associated gene expression during the progression of tumor metastasis. Because coexpression modules probably correspond to biological pathways, we focused on coexpression modules that are associated with a high number of protein-coding genes. Furthermore, NCBI RefSeq details the functions of many genes, which aided our identification of GC-associated genes. Using this method, we characterized the role of miR-144 in the peritoneal metastatic foci of GC. In the cancer coexpression network, miR-144 is connected to 6 protein-coding genes that are involved in tumor growth and metastasis (Figure 1C).

### Regulatory role of miR-144 in gastric cancer metastasis

**Figure 2:**
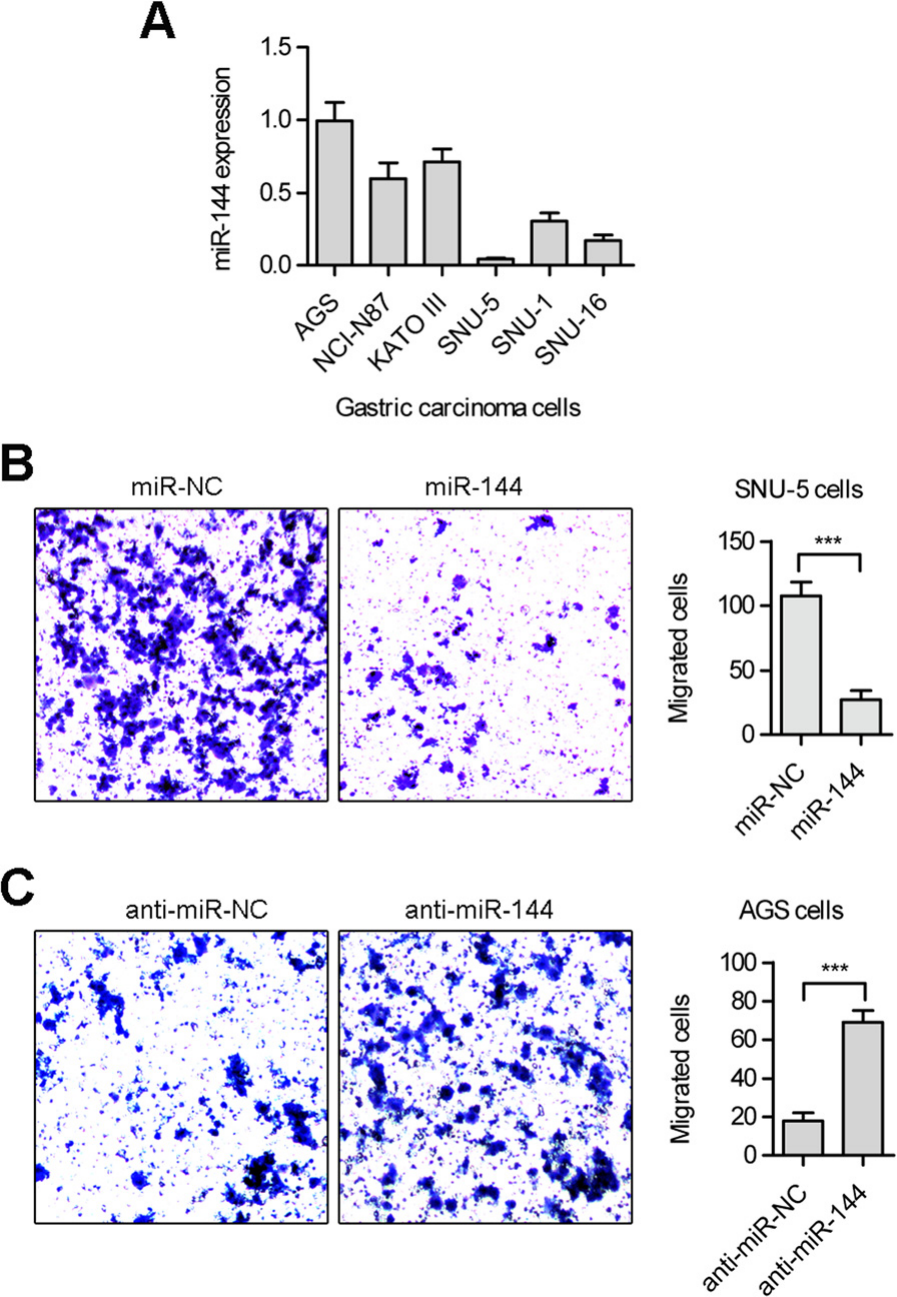
MiR-144 suppresses the migration of GC cells. **(A)** Expression levels of miR-144 were checked in a panel of 6 human GC cell lines using real-time PCR method. **(B)** Migration of SNU-5 cells treated with miR-144 mimics was checked using no-matrigel treated transwell chamber. **(C)** Migration of AGS cells treated with a miR-144 inhibitor was checked using no-matrigel treated transwell chamber. (***p < 0.001).

To investigate miR-144 function, we first examined miR-144 levels in a panel of 6 human GC cell lines. As shown in Figure 2A, we selected AGS, characterized with upregulated miR-144, and SNU-5, characterized with downregulated miR-144, for further study. The AGS cell line was derived from gastric tumor fragments that were resected from a patient who had received no prior therapy, while SNU-5 was derived from ascites of a patient with poorly differentiated carcinoma of the stomach.

In our study, we observed a close association between miR-144 loss and metastases in GC (Figure 1A and C). Correspondingly, earlier studies have documented miR-144-based inhibition of tumor cell migration and invasion in epithelial squamous cell carcinoma. We hypothesized that reintroduction of miR-144 expression would suppress cancer cell migration. Suitably, introduction of miR-144 expression inhibited cell migration in SNU-5 (Figure 2B). Compared to SNU-5, AGS cells have a relatively higher level of endogenous miR-144 expression. Unsurprisingly, inhibition of miR-144 increased AGS cell migration (Figure 2C). In fact, we also performed the invasion assay using matrigel-treated transwell, and there is no difference for invasive ability of SNU-5/AGS cells after treated with miR-144/anti-miR-144 (Data not shown). These results illustrated that miR-144 plays an important role in migration but not in invasion of GC cells.

### miR-144 affects MET expression

**Figure 3:**
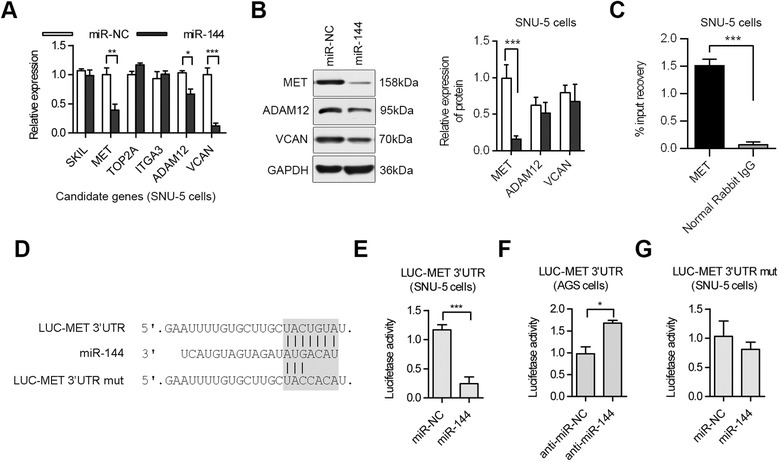
Expression of MET was regulated by miR-144. **(A)** mRNA levels of putative miR-144 targets were examined by real-time PCR in SNU-5 cells transfected with miR-144 mimics or miR-NC. **(B)** Protein levels of putative miR-144 targets were examined by western blotting in SNU-5 cells transfected with miR-144 mimics or miR-NC. **(C)** The % input recoveries of the RNA-ChIP reactions illustrates the enrichment by MET antibody. RNA-ChIP assay for miR-144 performed on anti-MET antibody from lysates of cells. RNA-ChIP with a nonrelated IgG served as controls. **(D)** A schematic picture of the predicted miR-144 binding site in the 3′UTR of MET. **(E)** SNU-5 cells were transiently co-transfected with LUC-MET 3′UTR and miR-144 mimic. **(F)** AGS cells were transiently co-transfected with LUC-MET 3′UTR and a miR-144 inhibitor. **(G)** Mutating of the miR-144 binding site in MET 3′UTR abolished the miR-144-induced luciferase activity suppression. Luciferase activity was measured after 24 h and normalised to the co-transfected Renilla. (*p < 0.05; **p < 0.01; ***p < 0.001).

Through the ectopic expression of miR-144 in SNU-5 cells, we determined ADAM12, VCAN and MET are putative miR-144 targets. As shown in Figure 3A, miR-144 expression dramatically affected the mRNA levels of ADAM12, VCAN and MET. ADAM12, VCAN and MET protein expression levels were also detected through western blot in cancer cells transfected with miR-144 mimics. As shown in Figure 3B, only MET protein levels were downregulated by miR-144. This indicates that miR-144 affects MET expression at the transcriptional level, perhaps through cleavage or destabilization of the mRNA structure. However, we also found the ADAM12 and VCAN protein levels are not decreased by exogenous introduction of miR-144. We thought that mRNA and protein levels cannot be directly correlated because of different half-life. We also thought they showed that may be due to the presence of miR-144 that was continuously repressing translation in one case but not the other. The MET:miR-144 interaction was also demonstrated in living cells by RNA-CHIP assay. In fact, endogenous miR-144 was found associated with endogenous MET in anti-MET but not anti-IgG immunoprecipitates from cells (Figure 3C).

Using bioinformatic-based analysis, we identified a single miRNA binding site for miR-144 in the 3′ UTR of MET mRNA (Figure 3D). To test if miR-144 directly binds the 3′-UTR of MET mRNA, we performed luciferase reporter assays in SNU-5 cells. PCR-derived fragments from MET 3′UTR were inserted into the pGL3 control vector at Xba1 site (LUC-MET 3′UTR). Co-transfection of LUC-MET 3′UTR and miR-144 mimics in SNU-5 cells resulted in a decreased luciferase signal (compared to miR-NC), confirming that binding of miR-144 to the 3′UTR of MET has a direct inhibitory effect (Figure 3E). The reverse experiment, accomplished by blocking endogenous miR-144 production with a miR-144 inhibitor in AGS cells, resulted in increased luciferase signal (Figure 3F). A mutated luciferase reporter at the miR-144 binding site was also constructed (Figure 3D). Mutation of the miRNA binding site abolished miR-144-mediated inhibition of luciferase activity (Figure 3G). These data suggest that the 1430–1436 position of the MET 3′UTR is critical for miR-144-mediated gene regulation.

### MET mediates the miR-144-induced resistance to migration

**Figure 4:**
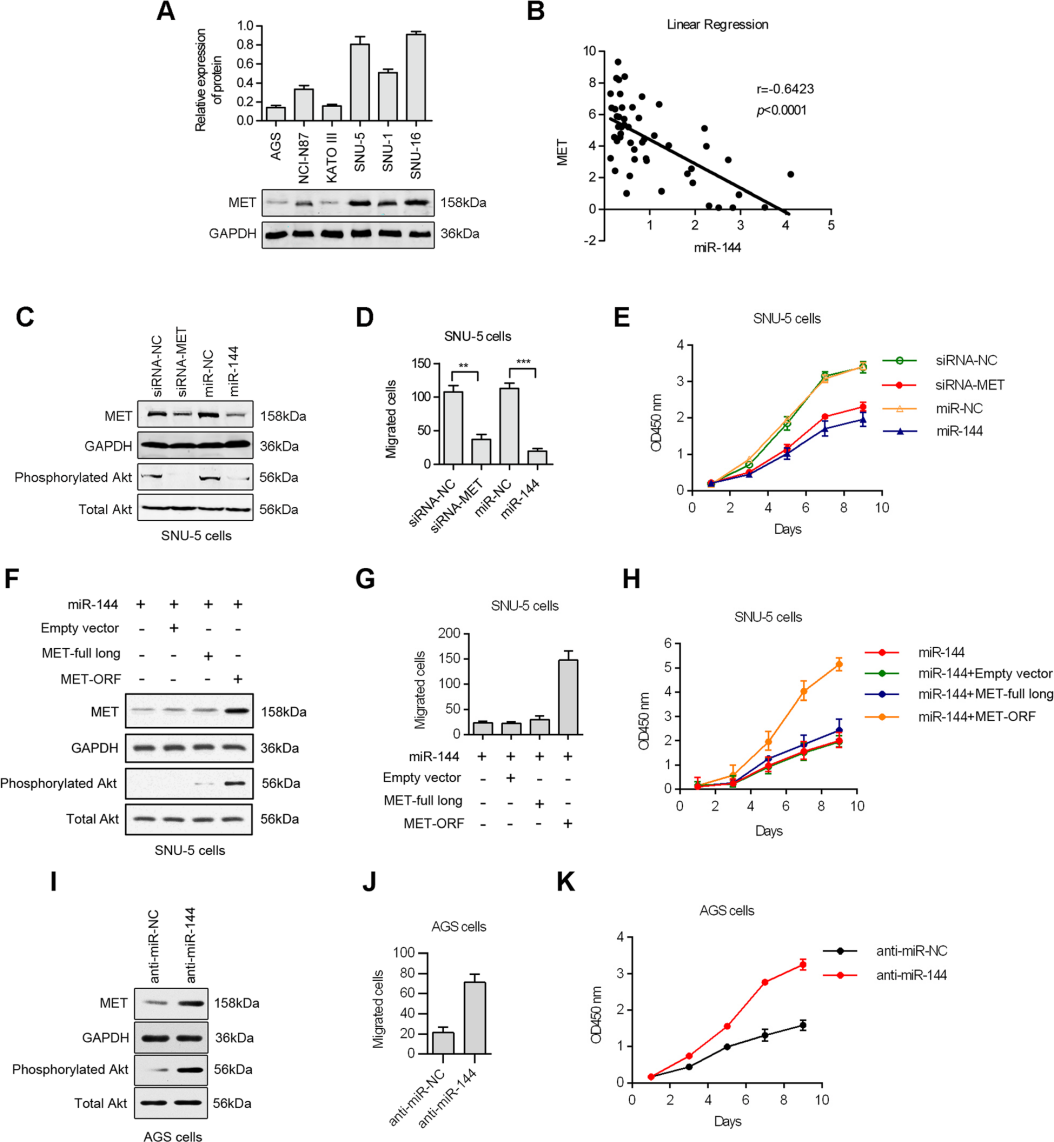
MET modulation accounts for the antimetastatic effect of miR-144. **(A)** Western blot showing MET expression in a set of human GC cell lines. **(B)** A significant inverse correlation is observed between the miR-144 and MET expression levels in the GC tissues (n = 52). **(C)** The MET and phosphorylated Akt were inhibited by the forced expression of miR-144 or siRNA-MET. **(D**, **E)** The effects of miR-144 or siRNA-MET on the migration and proliferation were determined in SNU-5 cells. **(F)** The MET and phosphorylated Akt were restored by the overexpression of MET in miR-144 mimics-treated SNU-5 cells. **(G**, **H)** The effects of miR-144 combined with MET-ORF on the migration and proliferation of SNU-5 cells. **(I)** The MET and phosphorylated Akt were up-regulated by blocking of miR-144 in AGS cells. **(J**, **K)** The effects of anti-miR-144 on the migration and proliferation of AGS cells.

Using real-time PCR, MET expression levels were determined for six human GC cell lines. As shown, cell lines with “downregulated” miR-144 levels have higher amounts of MET as compared to cell lines with “upregulated” miR-144 levels (Figure 4A). Using nonparametric tests, we determined a significant inverse correlation between MET mRNA and miR-144 expression in the GC metastatic samples (Figure 4B).

The MET protein functions as a receptor tyrosine kinase and plays a pivotal role in the promotion of cellular growth and migration by transducing extracellular stimuli to intracellular signaling circuits. A prominent component of the intracellular signaling machinery is the PI3K (phosphoinositide 3-kinase) pathway [20,21]. Because miR-144 inhibits MET expression, we hypothesized that miR-144 could ultimately decrease Akt phosphorylation and activation through decreased MET signaling. Accordingly, we examined Akt phosphorylation levels after miR-144 overexpression and observed a significant decrease of Akt phosphorylation (Figure 4C).

We decided to investigate whether miR-144-induced MET downregulation had an effect on tumor cell migration and proliferation. We transfected miR-144 and siRNA for MET (siRNA-MET) in SNU-5 cells. Cell migration was evaluated 16 h after transfection by transwell assay, while cell proliferation was determined through CCK-8. As shown in Figures 4D and E, transfection with miR-144 inhibited cell migration and proliferation as compared to control. Similarly, decreasing MET protein expression using siRNA also decreased tumor cell migration and proliferation.

To determine whether MET is the critical mediator of miR-144’s effect on cellular migration and proliferation, we constructed two MET expression vector. One of which contains only the open reading frame sequence of MET gene (MET-ORF), and another vector contains the full length nucleotide of MET gene including 3′UTR sequence (MET-full long). We then performed western blot analysis 48 h after transfection of MET-ORF/MET-full long into miR-144 mimics-treated SNU-5 cells (Figure 4F). Compared to the negative control group (Empty vector), ectopic expression of MET-ORF significantly increased the total expression of MET and phosphorylated Akt. Furthermore, expression of the MET-ORF promoted migration and proliferation of GC cells (Figure 4G and H). Overexpression of MET abolished miR-144-induced inhibition of cell migration and proliferation. In contrast, the protein level of MET and phosphorylated-AKT increased in AGS cells treated with anti-miR-144 (Figure 4I), and blocking of miR-144 also promoted the migration and proliferation of AGS cells (Figure 4J and K). These results indicate that MET is a critical target for the anti-migration effect of miR-144 in human GC cells.

## Discussion

In this study, we have identified non-overlapping signatures of a small number of miRNAs and genes that are aberrantly expressed in peritoneal metastatic tissues of GC, as compared to paired primary tissues. Analysis of miRNA and gene expression profiles in the miRNA-gene-network identified miR-144 as a regulator of major oncogenic pathways, such as proliferation and migration. GC patients with peritoneal metastases had lower miR-144 expression levels than patients without metastases. This finding implicates miR-144 as a potential tumor suppressor in GC. Furthermore, miR-144 is associated with the mechanisms of metastasis. Our results correspond with the results of earlier studies on the role of miR-144 in cancer proliferation, migration, and invasion [22,23]. miR-144 inhibits cancer cell metastasis by targeting the A disintegrin and metalloproteinase (ADAM) protein family member ADAMTS5. MicroRNA dysregulation is associated with increased tumor invasiveness and metastasis, as well as reduced patient prognosis in certain epithelial cancers [24]. We further investigated the role of miR-144 deregulation in GC. We studied the effect of miR-144 expression in the SNU-5 cell line, as it is characterized by low expression of miR-144. Ectopic expression of miR-144 in SNU-5 cells results in profound phenotypic changes, such as decreased migration. The reverse experiment, blocking miR-144 expression, was conducted in the AGS cell line, which has a relatively high endogenous level of miR-144 expression. Inhibition of miR-144 expression resulted in increased AGS cell migration.

To investigate the mechanism behind miR-144-dependent decreased migration of GC, we identified the putative targets for miR-144 as predicted by miRNA-gene-network. miR-144 overexpression can reduce MET expression at both mRNA and protein levels, and correspondingly, luciferase reporter assays revealed that miR-144 can directly interact with the MET 3′UTR. We then evaluated MET expression levels in a cohort of 52 GC patients and found that miR-144 levels are inversely correlated to MET expression. For that reason, we hypothesized that miR-144 inhibits GC tumorigenesis by targeting MET expression. MET has also been described as a miR-34a/c target in other cellular models and is known to promote motility and the invasive capability of tumor cells. Overexpression of MET is closely correlated with tumor invasion and patient prognosis in GC [6]. In GC, MET overexpression is an independent prognostic factor and potential drug target. Furthermore, MET overexpression can predict which patients may benefit from targeted therapy with MET inhibitors [25]. In our study, MET expression was significantly associated with GC differentiation, TNM and metastasis [26]. We determined that changes in cell proliferation and migration through miR-144 could be exerted through the regulation of MET expression. miR-144 repression leads to increased levels of MET, which may explain the metastasis phenotype of miR-144-depleted cells. Interestingly, miR-144 influenced hepatocyte growth factor (HGF) signaling. HGF, as the ligand of MET, can induce the activation of MET in epithelial cells. While MET overexpression could not completely restore GC tumorigenic qualities, GC cell migration and proliferation were partially restored after MET overexpression. Therefore, miR-144 may regulate other genes in GC cells. Previous studies have shown that MET can induce GC tumorigenesis through the activation of the PI3K pathway. In this study, we found that miR-144 significantly attenuated Akt phosphorylation, and that Akt phosphorylation was completely restored with overexpression of MET. Our findings suggest that miR-144 regulates Akt phosphorylation through MET regulation in GC.

In conclusion, our study identified a basis for the decreased level of miR-144 seen in GC metastatic tissues. miR-144 was identified as a potential tumor suppressor in GC and has been associated with the mechanisms of GC metastasis. Furthermore, miR-144 inhibits GC tumorigenesis by targeting MET, and subsequently, the PI3K/Akt pathway. To our knowledge, this is the first time miR-144 has been shown to target MET in GC cells. Therefore, further studies exploring the anticancer role of miR-144 may contribute to the development of new therapeutic strategies for GC.
